# Quasi-rerandomization for observational studies

**DOI:** 10.1186/s12874-023-01977-7

**Published:** 2023-06-30

**Authors:** Hengtao Zhang, Wen Su, Guosheng Yin

**Affiliations:** Department of Statistics and Actuarial Science, The University of Hong Kong, Hong Kong, China

**Keywords:** Causal inference, Covariate balance, Observational data, Rerandomization, Treatment effect

## Abstract

**Background:**

In the causal analysis of observational studies, covariates should be carefully balanced to approximate a randomized experiment. Numerous covariate balancing methods have been proposed for this purpose. However, it is often unclear what type of randomized experiments the balancing approaches aim to approximate; and this may cause ambiguity and hamper the synthesis of balancing characteristics within randomized experiments.

**Methods:**

Randomized experiments based on rerandomization, known for significant improvement on covariate balance, have recently gained attention in the literature, but no attempt has been made to integrate this scheme into observational studies for improving covariate balance. Motivated by the above concerns, we propose quasi-rerandomization, a novel reweighting method, where observational covariates are rerandomized to be the anchor for reweighting such that the balanced covariates obtained from rerandomization can be reconstructed by the weighted data.

**Results:**

Through extensive numerical studies, not only does our approach demonstrate similar covariate balance and comparable estimation precision of treatment effect to rerandomization in many situations, but it also exhibits advantages over other balancing techniques in inferring the treatment effect.

**Conclusion:**

Our quasi-rerandomization method can approximate the rerandomized experiments well in terms of improving the covariate balance and the precision of treatment effect estimation. Furthermore, our approach shows competitive performance compared with other weighting and matching methods. The codes for the numerical studies are available at https://github.com/BobZhangHT/QReR.

## Background

Randomized experiments are widely recognized as the gold standard for causal inference, due to the covariate balance and objectivity in treatment assignment [1, 2]. However, randomized experiments may be infeasible due to financial or ethical reasons, and it is often costly and takes a long time to conduct such experiments that may delay decision making. There is a tendency of using real-world evidence in observational studies with non-randomized data to infer the causal treatment effect in practice.

To analyze observational data, Bind and Rubin [3] recently advocated embedding the observational study in the context of a hypothetical randomized experiment, motivated by the ideas in [1, 4]. Specifically, they divided the analysis procedure into four major stages: (1) a *conceptual* stage that formulates the causal questions with the related assumptions in terms of a hypothetical randomized experiment; (2) a *design* stage that reconstructs the hypothetical randomized experiment on the observed data without access to the outcome data; (3) a *statistical analysis* stage that estimates the causal effect; and (4) a *summary* stage that summarizes the findings for the causal question.

In the cardinal design stage, many approaches have been proposed to approximate randomized experiments in terms of the covariate balance and thus reduce the estimation bias of treatment effect. One popular strategy uses a matching procedure, which generally assembles the units with similar propensity scores [5] between the treatment and control groups, including but not limited to the nearest-neighbor matching [6], optimal matching [7] and full matching [8]. One can refer to [9] for a systematic review of various matching approaches. Another scheme reweights observations to improve the covariate balance. One leading paradigm of reweighting is based on the inverse of propensity scores [10]. Imai and Ratkovic [11] leveraged the dual properties of propensity scores as a covariate balancing score to reweight samples. Hainmueller [12], Zubizarreta [13] and Chan et al. [14] directly optimized the sample weights to attain a set of predefined balancing conditions.

Although the aforementioned methods can improve the covariate balance, they cannot formally specify which type of randomized experiment is approximated. Such ambiguity would conceptually diminish the credibility of the whole observational analysis, because the experiment reconstructed at the design stage may not agree with the one considered at the conceptual stage. Furthermore, it may technically hinder the existing balancing approaches from synthesizing the valuable characteristics of some randomized experiments. Particularly, the celebrated rerandomization (ReR) proposed by Morgan and Rubin [15] has been widely recognized to outperform the classical complete randomization (CR) in terms of covariate balance [16]. It is thus highly preferable to make the balanced data approximate the rerandomized experiment rather than the CR experiment. Branson [17] proposed a test to diagnose the covariate balance of a matched dataset in contrast with rerandomzation, rather than directly balancing the observational data. Although the effectiveness of ReR has provoked further research for accommodating more complex experiments [18–20] and high-dimensional covariates [21–23], none of those extensions formally considered integrating rerandomization into the observational data analysis.

To address the above concerns, it is worth noting that only covariates are required for randomization. Therefore, we can also randomize the covariates in an observational study and then leverage the randomized data as the template to adjust the observational data. In this way, we bridge the observational study with the nominal randomized experiment, where adjusted observational data can directly imitate the appealing balancing properties. Towards this goal, we propose a reweighting approach, called quasi-rerandomization (QReR), which learns a generative neural network to yield random weight vectors such that the corresponding weighted datasets possess similar virtues of covariate balance to the rerandomized datasets.

Our approach has several advantages at the statistical analysis stage. First, our weight vectors can be conveniently paired with any weighted estimator for estimating the treatment effect. Second, it is allowed to ensemble multiple diverse weight vectors for improving estimation precision. Empirically, we compare the proposed method with the original rerandomization and other balancing methods through extensive numerical experiments. Not only does QReR demonstrate similar covariate balance and estimation performance to rerandomization in many situations, but it also shows superiority in estimating treatment effect in comparison with other balancing approaches, especially under the setting with complex response surfaces.

The remainder of this paper is organized as follows. In ‘Methods’, we introduce the problem setup of causal inference in observational studies and review the fundamental concepts of rerandomization. In particular, the subsection ‘Quasi-Rerandomization’ introduces the proposed QReR method in detail. In ‘Experiments and Discussion’, we conduct simulated experiments to compare our approach with rerandomization and other balancing algorithms as well as demonstrate the feasibility of our method with a real data example. We conclude with a discussion in ‘Conclusions’.

## Methods

### Treatment effect in observational studies

Suppose that the observational data consist of *N* units. Let $\varvec{T}=(T_1,\dots ,T_N)\in \{0,1\}^{N}$ denote the treatment allocation vector for all units, where $T_i=1$ if the *i*th unit is assigned to the treatment group and $T_i=0$ if it is assigned to the control group. We define $N_1=\sum _{i=1}^NT_i$ and $N_0=\sum _{i=1}^N(1-T_i)$ to be the corresponding sample sizes for treatment and control groups. Let $\varvec{X}_i=(X_{i1},\dots ,X_{id})^{\top }\in \mathbb {R}^{d}$ be the observed covariates for the *i*th unit. Following the potential outcome framework [24], each unit is associated with two potential outcomes $Y_i(0)$ and $Y_i(1)$ but only one of them can be observed,

$$\begin{aligned} Y_i^{\textrm{obs}}=Y_i(T_i)=T_iY_i(1)+(1-T_i)Y_i(0). \end{aligned}$$

It is typically assumed that there is no interference of the treatment effect between units and no hidden versions of treatment, known as the stable unit treatment value assumption (SUTVA) [25]. Furthermore, we impose the strongly ignorable assumption [5], such that the treatment assignment is independent of potential outcomes given the observed covariates and each unit has a chance to receive the treatment.

Given the samples $\{(\varvec{X}_i,Y_i^{\textrm{obs}},T_i)\}_{i=1}^N$, we aim to estimate the sample average treatment effect (SATE),

$$\begin{aligned} \tau _{\textrm{SATE}} = \frac{1}{N}\sum \limits _{i=1}^N\{Y_i(1)-Y_i(0)\}, \end{aligned}$$

which is also the causal estimand in rerandomization. In addition, we consider the estimation of the population average treatment effect (PATE),

$$\begin{aligned} \tau _{\textrm{PATE}} = \mathbb {E}(\tau _{\textrm{SATE}}) = \mathbb {E}\{Y(1)-Y(0)\}, \end{aligned}$$

where the population refers to the set from which the finite observations are sampled. The estimand $\tau _{\textrm{PATE}}$ is commonly considered in various balancing algorithms [9, 13, 14].

### Rerandomization

Given a covariate matrix $\varvec{X}=(\varvec{X}_1,\dots ,\varvec{X}_N)^{\top }\in \mathbb {R}^{N\times d}$, we elaborate on how a rerandomized experiment can be carried out to generate a balanced allocation and conduct inference. We first randomly generate a vector denoted by $\widetilde{\varvec{T}}=(\widetilde{T}_1,\dots ,\widetilde{T}_N)^{\top }\in \mathbb {R}^{N}$ with the constraint

1 $$\begin{aligned} \sum \limits _{i=1}^N\widetilde{T}_i=N_1,\quad \sum \limits _{i=1}^N(1-\widetilde{T}_i)=N_0. \end{aligned}$$

The covariate balance under the allocation $\widetilde{\varvec{T}}$ is then evaluated by the Mahalanobis distance,

2 $$\begin{aligned} D(\varvec{X},\widetilde{\varvec{T}})=\varvec{\Delta }(\widetilde{\varvec{T}})^{\top }\left[ \textrm{cov}\{\varvec{\Delta }(\widetilde{\varvec{T}})\}\right] ^{-1}\varvec{\Delta }(\widetilde{\varvec{T}})=\frac{N_1N_0}{N}\varvec{\Delta }(\widetilde{\varvec{T}})^{\top }\left\{ \widehat{\textrm{cov}}(\varvec{X})\right\} ^{-1}\varvec{\Delta }(\widetilde{\varvec{T}}), \end{aligned}$$

where the vector $\varvec{\Delta }(\widetilde{\varvec{T}}) = \bar{\varvec{X}}_1-\bar{\varvec{X}}_0$ is the mean difference of covariates between the treatment and control groups with $\bar{\varvec{X}}_1=\sum _{i=1}^N\widetilde{T}_i\varvec{X}_i/N_1$ and $\bar{\varvec{X}}_0=\sum _{i=1}^N(1-\widetilde{T}_i)\varvec{X}_i/N_0$. The matrix $\textrm{cov}\left\{ \varvec{\Delta }(\widetilde{\varvec{T}})\right\}$ refers to the covariance of $\varvec{\Delta }(\widetilde{\varvec{T}})$ regarding all random $\widetilde{\varvec{T}}$’s under the constraint (1), and $\widehat{\textrm{cov}}(\varvec{X})$ is the sample covariance matrix with respect to $\varvec{X}$. We accept the allocation $\widetilde{\varvec{T}}$ if the corresponding Mahalanobis distance is no larger than a prefixed threshold $a>0$, i.e., $D(\varvec{X},\widetilde{\varvec{T}})\le a$; otherwise a new allocation $\widetilde{\varvec{T}}$ is generated. Morgan and Rubin [15] showed that $D(\varvec{X},\widetilde{\varvec{T}})$ asymptotically follows a chi-squared distribution so that one can determine the threshold *a* by $P(\chi ^2_d\le a)=p_a$ given a predefined acceptance probability $p_a\in (0,1]$. A smaller $p_a$ used in rerandomization leads to more balanced covariates, and when $p_a=1$, rerandomization reduces to the complete randomization.

### Connecting rerandomization and observational studies

For the observational data analysis, the covariates $\varvec{X}_i$’s should be carefully balanced to approximate a hypothetical randomized experiment without access to the outcome variables. This stage helps to reduce the underlying confounding effects in the observational data for estimating $\tau _{\textrm{SATE}}$ or $\tau _{\textrm{PATE}}$. Particularly, most existing balancing techniques are proposed to essentially achieve some of the following empirical equations,

3 $$\begin{aligned} \sum \limits _{i=1}^{N}W_iT_if(\varvec{X}_i)= & {} \sum \limits _{i=1}^{N}W_i(1-T_i)f(\varvec{X}_i), \end{aligned}$$

4 $$\begin{aligned} \frac{1}{N}\sum \limits _{i=1}^{N}f(\varvec{X}_i)= & {} \sum \limits _{i=1}^{N}W_iT_if(\varvec{X}_i), \end{aligned}$$

5 $$\begin{aligned} \frac{1}{N}\sum \limits _{i=1}^{N}f(\varvec{X}_i)= & {} \sum \limits _{i=1}^{N}W_i(1-T_i)f(\varvec{X}_i). \end{aligned}$$

where $\varvec{W}=(W_1,\dots ,W_N)$ denotes the vector of sample weights, and *f* refers to an vector-valued function of $\varvec{X}_i$’s. Heuristically, the first equation directly ensures the covariate balance between treatment and control groups, which is typically leveraged by propensity-score-based approaches. The propensity score $\pi (\varvec{X}_i,\varvec{\eta })=P(T_i=1|\varvec{X}_i)$ can be estimated for each sample with unknown parameter $\varvec{\eta }$. It can be shown that the propensity scores satisfy $W_i=T_i/\pi (\varvec{X}_i,\varvec{\eta })+(1-T_i)/\{1-\pi (\varvec{X}_i,\varvec{\eta })\}$ and $f(\varvec{X}_i)=\partial \pi (\varvec{X}_i,\varvec{\eta })/\partial \varvec{\eta }$ with respect to (3) [11]. Equations (4) and (5) quantify the fact that randomized covariates in treatment and control groups have similar characteristics to the pooled covariates, which also imply the covariate balance in Eq. (3). Some balancing techniques hereby treat $W_i$’s as unknown parameters for optimization, and modify (4) and (5) as either hard equality constraints [12, 14] or soft constraints, namely $|\frac{1}{N}\sum _{i=1}^{N}f(\varvec{X}_i) -\sum _{i=1}^{N}W_iT_if(\varvec{X}_i)|\le \delta$ with a threshold $\delta >0$ [13]. The function $f(\cdot )$ is typically specified as the covariate moment, such as $f(\varvec{X}_i)=\varvec{X}_i$, in those constraints.

However, the aforementioned approaches do not clarify what type of randomized experiment (e.g., complete randomization, rerandomization, or constrained randomization) they intend to approximate, which not only leads to conceptual uncertainty, but can also prevent integrating the properties of randomized experiments into balancing the covariates. One salient potential of such integration is to directly and objectively calibrate the covariate balance based on properly randomized covariates without assuming a parametric propensity score model for (3) or completely depending on implicit balancing constraints in (4) and (5). Finally, one may also exploit the superior balance of some randomized experiments, such as rerandomization rather than complete randomization, to enhance efficiency and inference in observational studies.

We hence propose the quasi-rerandomization (QReR), a reweighting method, to bridge rerandomization and observational studies. We first conduct rerandomization over the covariates and generate multiple acceptable allocation vectors, because rerandomization does not require the availability of responses. We then compute a balancing metric based on (2) for all acceptable assignments. Those metric values imply how the covariates would be balanced under the rerandomized experiment, and thus can be adopted as the anchor to guide further balance adjustment based on (3), (4) and (5) for the observational data.

### Quasi-rerandomization

In quasi-rerandomization, we first generate a large number of acceptable rerandomized allocations. A transformation function is then fitted via a neural network, which generates weight vectors from Dirichlet noises such that the weighted covariates have comparable balance properties to the rerandomized covariates.

Specifically, the sample weights satisfy $\varvec{W}^{\top }\varvec{T}=1$ and $\varvec{W}^{\top }(\varvec{1}_N-\varvec{T})=1$, where $\varvec{1}_N\in \mathbb {R}^{N}$ is a vector of length *N* with all entries of 1, and $\varvec{T}=(T_1,\dots ,T_N)$ is the observed treatment allocation vector. Without loss of generality, we assume that $\varvec{T}=(\varvec{1}^{\top }_{N_1},\varvec{0}^{\top }_{N_0})^{\top }$, and the weight vector can thus be rewritten as $\varvec{W}=(\varvec{W}_1^{\top },\varvec{W}_0^{\top })^{\top }$, where $\varvec{W}_1$ and $\varvec{W}_0$ respectively denote the sub-vectors in $\varvec{W}$ for the treatment and control units with $\varvec{W}_1^{\top }\varvec{1}_{N_1}=1$ and $\varvec{W}_0^{\top }\varvec{1}_{N_0}=1$. We assume that $\varvec{W}_1$ and $\varvec{W}_0$ are drawn from Dirichlet distributions $\textrm{Dir}(\varvec{1}_{N_1})$ and $\textrm{Dir}(\varvec{1}_{N_0})$, respectively. We aim to learn a transformation function $\widetilde{\varvec{W}} = G(\varvec{W}|\varvec{X},\varvec{\theta })$ with parameter $\varvec{\theta }$ such that the weighted covariate mean difference with respect to $\widetilde{\varvec{W}}$,

$$\begin{aligned} \varvec{\Delta }(\widetilde{\varvec{W}}) = \sum \limits _{i=1}^N\widetilde{W}_{i}T_{i}\varvec{X}_{i}-\sum \limits _{i=1}^N\widetilde{W}_{i}(1-T_{i})\varvec{X}_{i}, \end{aligned}$$

has a similar distribution to $\varvec{\Delta }(\widetilde{\varvec{T}})$. Intuitively, we treat the rerandomized covariate mean difference $\varvec{\Delta }(\widetilde{\varvec{T}})$ as the template to adjust the weighted counterpart $\varvec{\Delta }(\widetilde{\varvec{W}})$, where $\varvec{\Delta }(\widetilde{\varvec{W}})$ corresponds to (3) with $f(\varvec{X}_i)=\varvec{X}_i$. As a result, the reweighted observational data imitate the balance characteristics as in the rerandomized data. We specify $G(\varvec{W}|\varvec{X},\varvec{\theta })$ as a multi-layer neural network. The neural network has two hidden layers with each layer containing 512 neurons, where the number of neurons is selected based on the empirical observation that this value is sufficient in most cases. We adopt the ReLU activation function [26] for both hidden layers. We also apply the dropout scheme [27] with a dropout rate of 0.5 after each hidden layer, which is a common choice as suggested by [28]. The output layer applies two separate softmax functions to yield the unified weight vector $\widetilde{\varvec{W}}=(\widetilde{\varvec{W}}_1^{\top },\widetilde{\varvec{W}}_0^{\top })^{\top }$.

The rationale for approximating the distribution of the covariate mean difference $\varvec{\Delta }(\widetilde{\varvec{T}})$ from rerandomization is given as follows. First, the vector $\varvec{\Delta }(\widetilde{\varvec{T}})$ itself is a balance measure, and thus the approximation can ensure the fundamental balancing property of our weighted data. Second, the distribution-based approximation can further incorporate the characteristics of covariate balance from rerandomization into the weighted data. In rerandomization, the covariate balance of all acceptable allocations is determined by the distribution of Mahalanobis distance, which is fully governed by $\varvec{\Delta }(\widetilde{\varvec{T}})$ because the sample covariance matrix $\widehat{\textrm{cov}}(\varvec{X})$ is fixed for the observed covariates as illustrated by (2). Moreover, the distribution of $\varvec{\Delta }(\widetilde{\varvec{T}})$ contains more abundant high-dimensional information among covariates for the balancing property in contrast to that of Mahalanobis distance. Finally, the distribution-based approximation can also provide randomness and diversity for our generated weight vectors, which offers more options for making inference at the statistical analysis stage, such as using some ensembling techniques to estimate the treatment effects. Specifically, one may follow the idea of bagging [29] by separately applying a weighted treatment effect estimator (e.g., the weighted mean difference of response and the doubly robust estimator [30]) to each weight vector and then aggregating all estimators by taking the mean or median.

Motivated by [31], we adopt the maximum mean discrepancy (MMD) proposed by [32, 33] as the loss function to minimize the distribution deviation between $\varvec{\Delta }(\widetilde{\varvec{W}})$ and $\varvec{\Delta }(\widetilde{\varvec{T}})$. Let $\{\varvec{\delta }(\widetilde{\varvec{T}}^{(b)})\}_{b=1}^B$ and $\{\varvec{\delta }(\widetilde{\varvec{W}}^{(b)})\}_{b=1}^B$ be the samples for loss calculation, where $\{\widetilde{\varvec{T}}^{(b)}\}_{b=1}^B$ are initially obtained from rerandomization, and $\{\widetilde{\varvec{W}}^{(b)}\}_{b=1}^B$ with $\widetilde{\varvec{W}}^{(b)}=G({\varvec{W}}^{(b)}|\varvec{X},\varvec{\theta })$ are obtained through transformation from the initial Dirichlet weights $\{{\varvec{W}}^{(b)}\}_{b=1}^B$. Based on those samples, the MMD loss is defined as

6 $$\begin{aligned} \mathcal {L}_{\textrm{MMD}}(\varvec{\theta })= & {} \left\| \frac{1}{B}\sum \limits _{b=1}^{B}\phi \left\{ \varvec{\delta }(\widetilde{\varvec{W}}^{(b)})\right\} -\frac{1}{B} \sum \limits _{b=1}^{B}\phi \left\{ \varvec{\delta }(\widetilde{\varvec{T}}^{(b)})\right\} \right\| _2 \nonumber \\= & {} \left[ \frac{1}{B^2}\sum \limits _{i=1}^{B}\sum \limits _{j=1}^{B}\phi \left\{ \varvec{\delta }(\widetilde{\varvec{W}}^{(i)})\right\} ^{\top } \phi \left\{ \varvec{\delta }(\widetilde{\varvec{W}}^{(j)})\right\} \right. \nonumber \\{} & {} +\frac{1}{B^2}\sum \limits _{i=1}^{B}\sum \limits _{j=1}^{B}\phi \left\{ \varvec{\delta }(\widetilde{\varvec{T}}^{(i)})\right\} ^{\top } \phi \left\{ \varvec{\delta }(\widetilde{\varvec{T}}^{(j)})\right\} \nonumber \\{} & {} \left. -\frac{2}{B^2}\sum \limits _{i=1}^{B}\sum \limits _{j=1}^{B}\phi \left\{ \varvec{\delta }(\widetilde{\varvec{W}}^{(i)})\right\} ^{\top } \phi \left\{ \varvec{\delta }(\widetilde{\varvec{T}}^{(j)})\right\} \right] ^{1/2} \nonumber \\= & {} \left[ \frac{1}{B^2}\sum \limits _{i=1}^{B}\sum \limits _{j=1}^{B}K\left\{ \varvec{\delta }(\widetilde{\varvec{W}}^{(i)}), \varvec{\delta }(\widetilde{\varvec{W}}^{(j)})\right\} \right. \nonumber \\{} & {} +\frac{1}{B^2}\sum \limits _{i=1}^{B}\sum \limits _{j=1}^{B}K\left\{ \varvec{\delta }(\widetilde{\varvec{T}}^{(i)}), \varvec{\delta }(\widetilde{\varvec{T}}^{(j)})\right\} \nonumber \\{} & {} \left. -\frac{2}{B^2}\sum \limits _{i=1}^{B}\sum \limits _{j=1}^{B}K\left\{ \varvec{\delta }(\widetilde{\varvec{W}}^{(i)}), \varvec{\delta }(\widetilde{\varvec{T}}^{(j)})\right\} \right] ^{1/2}, \end{aligned}$$

where $\phi (\varvec{x})$ is the feature mapping vector, and $K(\varvec{x},\varvec{y})=\phi (\varvec{x})^{\top }\phi (\varvec{y})$ is the corresponding kernel function with respect to any feature vectors $\varvec{x}, \varvec{y}$.

If $\phi$ is the identity mapping, the MMD loss reduces to the Euclidean norm of the difference between sample means of $\{\varvec{\delta }(\widetilde{\varvec{T}}^{(b)})\}_{b=1}^B$ and $\{\varvec{\delta }(\widetilde{\varvec{W}}^{(b)})\}_{b=1}^B$, i.e., the deviation between the first moments. Using the kernel trick, one can implicitly map the sample vectors to a high-dimensional and nonlinear feature space, and the loss would correspond to the difference between higher-order moments of two samples. Gretton et al. [32, 33] showed that when the feature space is a universal reproduced kernel Hilbert space, the MMD loss asymptotically equals zero if and only if the distributions of $\varvec{\Delta }(\widetilde{\varvec{T}})$ and $\varvec{\Delta }(\widetilde{\varvec{W}})$ are the same. We choose the common radial basis function (RBF) kernel $K(\varvec{x}_1,\varvec{x}_2)=\exp (- \gamma \Vert \varvec{x}_1-\varvec{x}_2\Vert ^2_2)$ with bandwidth $\gamma$, whose feature space consists of moments of all orders.

We incorporate two additional regularization terms to the MMD loss to further improve the balancing and inference properties for the generated weights. It is easy to derive the relationships concerning any allocation vector ${\varvec{T}}$ between the fixed sample mean $\bar{\varvec{X}}=\sum _{i=1}^N\varvec{X}_i/N$ and the sample means of treatment and control groups, $\bar{\varvec{X}}_1$ and $\bar{\varvec{X}}_0$,

$$\begin{aligned} \varvec{\delta }(\varvec{T}) = \frac{N}{N_0}\left( \bar{\varvec{X}}_1-\bar{\varvec{X}}\right) = -\frac{N}{N_1}\left( \bar{\varvec{X}}_0-\bar{\varvec{X}}\right) . \end{aligned}$$

Thus, the balance measure $\varvec{\delta }(\varvec{T})$ can be represented by the mean difference $\bar{\varvec{X}}-\bar{\varvec{X}}_1$ or $\bar{\varvec{X}}-\bar{\varvec{X}}_0$. Moreover, $\bar{\varvec{X}}_1$ and $\bar{\varvec{X}}_0$ are close to the fixed $\bar{\varvec{X}}$ under the rerandomization due to $\varvec{\delta }(\widetilde{\varvec{T}})\approx \varvec{0}$ when $\varvec{T}=\widetilde{\varvec{T}}$, i.e., $\bar{\varvec{X}}\approx \bar{\varvec{X}}_1\approx \bar{\varvec{X}}_0$. The first regularizer aims to retain the above characteristics of rerandomization for QReR,

7 $$\begin{aligned} \mathcal {R}_1(\varvec{\theta }) =\frac{1}{B}\sum \limits _{b=1}^B\left( \left\| \sum \limits _{j=1}^N\widetilde{W}^{(b)}_{j}T_{j}\varvec{X}_{j}-\bar{\varvec{X}}\right\| _2^2+\left\| \sum \limits _{j=1}^N\widetilde{W}^{(b)}_{j}(1-T_{j})\varvec{X}_{j}-\bar{\varvec{X}}\right\| _2^2\right) . \end{aligned}$$

This regularizer essentially ensures the Eqs. in (4) and (5) for constraining the imbalance among covariates. To avoid extreme values among the weights, we introduce another regularization term,

8 $$\begin{aligned} \mathcal {R}_2(\varvec{\theta }) = \frac{1}{B}\sum \limits _{b=1}^B\left( \left\| \widetilde{\varvec{W}}_1^{(b)}-\varvec{1}_{N_1}/N_1\right\| _2^2 +\left\| \widetilde{\varvec{W}}_0^{(b)}-\varvec{1}_{N_0}/N_0\right\| _2^2\right) . \end{aligned}$$

Modified from the objective function of the stable balancing weights [13], this regularizer can control the variation of the transformed weights within each treatment group and help to avoid extreme weights, which can further improve inference, such as reducing the variance of the weighted point estimator. The uniform weights $\varvec{1}_{N_1}/N_1$ and $\varvec{1}_{N_0}/N_0$ are widely used as the base weights [12–14]. Based on (7) and (8), we estimate the parameter $\varvec{\theta }$ of the network model by solving the optimization problem,

9 $$\begin{aligned} \min _{\varvec{\theta }} \left\{ \mathcal {L}_{\textrm{MMD}}(\varvec{\theta }) + \lambda _1\mathcal {R}_1(\varvec{\theta }) + \lambda _2\mathcal {R}_2(\varvec{\theta })\right\} , \end{aligned}$$

where $\lambda _1$ and $\lambda _2$ are the positive regularization parameters.

### Network training

The network training can be divided into three main steps: network initialization, loss computation with parameter $\varvec{\theta }$ updating, and early stopping of the training, as detailed in Algorithm 1. Accordingly, three distinct sets of weight vectors and acceptable ReR allocations are generated in advance for implementing those steps. We generate $B_{\textrm{init}}$ weight vectors $\{\varvec{W}^{(b)}\}_{b=1}^{B_{\textrm{init}}}$ for network initialization, $B_{\textrm{loss}}$ acceptable allocations $\{\widetilde{\varvec{T}}^{(b)}\}_{b=1}^{B_{\textrm{loss}}}$ for loss computation as well as parameter updating, and $B_{\textrm{stop}}$ weight vectors $\{\varvec{W}^{(b)}\}_{b=1}^{B_{\textrm{stop}}}$ together with allocations $\{\widetilde{\varvec{T}}^{(b)}\}_{b=1}^{B_{\textrm{stop}}}$ for early stopping.

**Figure:**
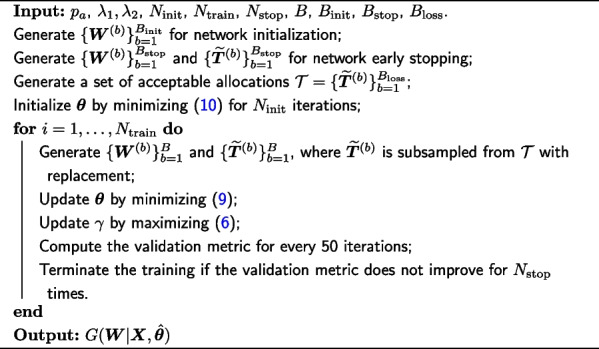
**Algorithm 1** Network Training Algorithm.

In the network initialization for the parameter $\varvec{\theta }$, we minimize the mean squared error between the logarithmic weights $\{\log (\varvec{W}^{(b)})\}_{b=1}^{B_{\textrm{init}}}$ and $\{\log (\widetilde{\varvec{W}}^{(b)})\}_{b=1}^{B_{\textrm{init}}}$ with $\widetilde{\varvec{W}}^{(b)}=G({\varvec{W}}^{(b)}|\varvec{X},\varvec{\theta })$,

10 $$\begin{aligned} \min _{\varvec{\theta }}\frac{1}{B_{\textrm{init}}}\sum \limits _{b=1}^{B_{\textrm{init}}}\left\| \log (\varvec{W}^{(b)})-\log (\widetilde{\varvec{W}}^{(b)})\right\| _2^2, \end{aligned}$$

where the logarithmic transformation helps to amplify the difference between vectors. The intuition is that the initial network should be close to the identity mapping, i.e., $\varvec{W}\approx G(\varvec{W}|\varvec{X},\varvec{\theta })$. Our initialization can be viewed as a special type of unsupervised pre-training [28, 34], which acts as a regularizer to improve the robustness of the network [35].

After initializing the network, we calculate the loss and optimize the network parameter $\varvec{\theta }$ using the stochastic gradient descent. Different from the typical network training with a prefixed dataset, our training data, $\varvec{\delta }(\widetilde{\varvec{T}})$ and $\varvec{\delta }(\widetilde{\varvec{W}})$, can be generated infinitely from rerandomization and Dirichlet distributions; that is, we can generate a new batch of $\widetilde{\varvec{T}}^{(b)}$’s and $\varvec{W}^{(b)}$’s for every iteration. However, it could be computationally expensive to generate new $\widetilde{\varvec{T}}^{(b)}$’s in each iteration if the acceptance probability $p_a$ for rerandomization is small. We thus generate a large number of feasible allocations $\{\widetilde{\varvec{T}}^{(b)}\}_{b=1}^{B_{\textrm{loss}}}$ prior to the network training, and then sample a small subset $\{\widetilde{\varvec{T}}^{(b)}\}_{b=1}^B$ from $\{\widetilde{\varvec{T}}^{(b)}\}_{b=1}^{B_{\textrm{loss}}}$ with replacement. A new batch of weight vectors $\{\widetilde{\varvec{W}}^{(b)}\}_{b=1}^B$ are jointly generated for the loss calculation.

Given a bandwidth $\gamma$ and the regularization coefficients $\lambda _1$ and $\lambda _2$, we minimize the loss function (9) to update $\varvec{\theta }$ based on $\{\varvec{\delta }(\widetilde{\varvec{T}}^{(b)})\}_{b=1}^B$ and $\{\varvec{\delta }(\widetilde{\varvec{W}}^{(b)})\}_{b=1}^B$ for each training iteration. The regularization parameters $\lambda _1$ and $\lambda _2$ are kept as constants throughout the training, while the bandwidth parameter $\gamma \in (0,\infty )$ should be properly chosen to maximize the MMD loss $\mathcal {L}_{\textrm{MMD}}(\varvec{\theta })$ [31, 36]. Instead of fixing the value of $\gamma$ [31] or conducting a heuristic line search for $\gamma$ [36], we adopt a data-driven strategy to adaptively update the value of $\gamma$ along with the network training through the stochastic gradient descent. Given the updated network parameter $\varvec{\theta }$, we update $\gamma$ to maximize the MMD loss (6) based on the same batch of weight vectors and ReR allocations.

We also introduce a metric based on the Mahalanobis distance to early stop the training once the metric cannot be improved for more iterations. We define a weighted Mahalanobis distance $D(\varvec{X},\widetilde{\varvec{W}})$ for the vector $\widetilde{\varvec{W}}$ by plugging the corresponding weighted mean and covariance estimators into (2),

11 $$\begin{aligned} D(\varvec{X},\widetilde{\varvec{W}}) = \frac{N_1N_0}{N}\varvec{\Delta }(\widetilde{\varvec{W}})^{\top }\left\{ \widehat{\textrm{cov}}_{\widetilde{\varvec{W}}}(\varvec{X})\right\} ^{-1}\varvec{\Delta }(\widetilde{\varvec{W}}), \end{aligned}$$

where

$$\begin{aligned} \widehat{\textrm{cov}}_{\widetilde{\varvec{W}}}(\varvec{X})=\frac{1}{1-\sum _{i=1}^N\widetilde{W}^{*2}_i}\sum \limits _{i=1}^N \widetilde{W}^*_i(\varvec{X}_i-\bar{\varvec{X}}_{\widetilde{\varvec{W}}})(\varvec{X}_i-\bar{\varvec{X}}_{\widetilde{\varvec{W}}})^{\top }, \end{aligned}$$

with

$$\begin{aligned} \bar{\varvec{X}}_{\widetilde{\varvec{W}}} = \sum \limits _{i=1}^N\widetilde{W}^*_i\varvec{X}_i\quad \textrm{and} \quad \widetilde{W}^*_i = \left\{ N_1T_i+N_0(1-T_i)\right\} \widetilde{W}_i/N, \quad i =1,\dots , N. \end{aligned}$$

It is easy to check that $\widehat{\textrm{cov}}_{\widetilde{\varvec{W}}}(\varvec{X}) = \widehat{\textrm{cov}}(\varvec{X})$ and $D(\varvec{X},\widetilde{\varvec{W}})=D(\varvec{X},\widetilde{\varvec{T}})$ when the weights are all equal within the treatment and the control groups, i.e., $\widetilde{\varvec{W}}_1=\varvec{1}_{N_1}/N_1$ and $\widetilde{\varvec{W}}_0=\varvec{1}_{N_0}/N_0$. Motivated by the test proposed in [17], where the distribution of Mahalanobis distance incorporates the characteristics of covariate balance for randomized experiments, we specify the stopping metric as the Kolmogorov–Smirnov statistic with respect to the empirical distributions of Mahalanobis distances $\{D(\varvec{X},\widetilde{\varvec{W}}^{(b)})\}_{b=1}^{B_{\textrm{stop}}}$ and $\{D(\varvec{X},\widetilde{\varvec{T}}^{(b)})\}_{b=1}^{B_{\textrm{stop}}}$ based on $\{\varvec{W}^{(b)}\}_{b=1}^{B_{\textrm{stop}}}$ and $\{\widetilde{\varvec{T}}^{(b)}\}_{b=1}^{B_{\textrm{stop}}}$ respectively. Heuristically, a smaller value of the stopping metric indicates that the weighted data obtained from our model is more likely to resemble the rerandomized experiment.

### Estimation for weighted data

After the network training, one can conduct statistical analysis using a set of transformed weights $\{\widetilde{\varvec{W}}^{(m)}\}_{m=1}^M$ generated from the trained network, where *M* denotes the number of weight vectors used for estimation. For the sample average treatment effect $\tau _{\textrm{SATE}}$, we adopt the mean difference estimator,

12 $$\begin{aligned} \hat{\tau }(\widetilde{\varvec{W}},\varvec{Y}^{\textrm{obs}}) = \sum \limits _{i=1}^N\widetilde{W}_{i}T_{i}Y^{\textrm{obs}}_{i}-\sum \limits _{i=1}^N\widetilde{W}_{i}(1-T_{i})Y^{\textrm{obs}}_{i}. \end{aligned}$$

Given $\{\hat{\tau }(\widetilde{\varvec{W}}^{(m)},\varvec{Y}^{\textrm{obs}})\}_{m=1}^M$, we consider the following point estimator for $\tau _{\textrm{SATE}}$,

13 $$\begin{aligned} \hat{\tau }_{M} = \frac{1}{M}\sum \limits _{m=1}^{M}\hat{\tau }(\widetilde{\varvec{W}}^{(m)},\varvec{Y}^{\textrm{obs}}) = \hat{\tau }\left( \frac{1}{M}\sum \limits _{m=1}^M\widetilde{\varvec{W}}^{(m)},\varvec{Y}^{\textrm{obs}}\right) . \end{aligned}$$

Such an estimation strategy leverages multiple weight vectors, and is equivalent to a weighted mean difference estimator based on the average weight vector $\sum _{m=1}^M\widetilde{\varvec{W}}^{(m)}/M$. When using one vector with $M=1$, the estimator mimics the estimation in rerandomization, where only one acceptable allocation would be used to collect the responses and estimate $\tau _{\textrm{SATE}}$.

For a single weight vector $\widetilde{\varvec{W}}$, we can pass it to any point estimator designed for $\tau _{\textrm{PATE}}$ that supports weighted inputs, and the confidence interval can be constructed accordingly based on the robust sampling variance of the weighted point estimator [12, 37]. Particularly, we consider the simplest estimator using a weighted linear model to regress responses on the allocation indicators. Through some linear algebras, it is easy to check that such a point estimator for $\tau _{\textrm{PATE}}$ exactly has the same form of the weighted mean difference in (12), and we can simultaneously obtain its sampling variance based on the linear model. Therefore, we similarly consider the ensembled estimator $\hat{\tau }_{M}$ based on the average weight vector, where the aggregated vector empirically leads to smaller bias and root mean squared error than a random single vector.

## Experiments and discussion

### Experimental settings

The simulated settings are modified from those in [17, 38], which are designed to mimic the real cases. We fix the sample size for the treatment group as $N_1=250$ and set the sample size of the control group as $N_0=r\times N_1$ with the ratio $r\in \{1, 2\}$. The treatment indicator vector is kept as $\varvec{T}=(\varvec{1}_{N_1}^{\top },\varvec{0}_{N_0}^{\top })^{\top }$ for a given ratio *r*. The 8-dimensional covariate vector $\varvec{X}_i=(X_{i1},\dots ,X_{i8})^{\top }$ is simulated as follows,

$$\begin{aligned} (X_{i1},\dots ,X_{i4})|T_i&\sim N\left( T_i\varvec{\mu },T_i\varvec{\Sigma }+(1-T_i)\varvec{I}_4\right) ,\\ X_{i5}, X_{i6}|T_i&\sim \textrm{Bernoulli}(0.1+0.068T_i),\\ X_{i7}, X_{i8}|T_i&\sim \textrm{Bernoulli}(0.4+0.242T_i),\quad i=1,\dots ,N, \end{aligned}$$

where we consider three different combinations of $\varvec{\mu }$ and $\varvec{\Sigma }$,

- Scenario 1: $$\varvec{\mu }=(0.2,0.2,0.5,0.5)^{\top }$$ and $$\varvec{\Sigma }=\varvec{I}_4$$;
- Scenario 2: $$\varvec{\mu }=\sqrt{1.5}\times (0.2,0.2,0.5,0.5)^{\top }$$ and $$\varvec{\Sigma }=2\varvec{I}_4$$;
- Scenario 3: $$\varvec{\mu }=\sqrt{1.5}\times (0.2,0.2,0.5,0.5)^{\top }$$ and $$\varvec{\Sigma }=1.5\varvec{I}_4+0.5\varvec{1}_4\varvec{1}_4^{\top }$$.

Scenarios 1 and 2 represent the cases where the continuous covariates respectively have homogeneous and heterogeneous variances between the treatment and control groups. Scenario 3 considers the situation where there exist correlations among the Gaussian covariates in the treatment group. Let $(\mu _{T}-\mu _{C})/\sqrt{(\sigma ^2_T+\sigma ^2_C)/2}$ and $(p_{T}-p_{C})/\sqrt{\{p_T(1-p_T)+p_C(1-p_C)\}/2}$ be the true standardized mean difference for the Gaussian and Bernoulli covariates respectively, where $\mu _{}$ and $\sigma ^2$ correspond to the true mean and variance for a Gaussian variable, and *p* is the probability of taking a value of 1 for a Bernoulli variable. In all three scenarios, we keep the true standardized mean difference as 0.2 or 0.5 for each covariate, which represents a meaningful covariate imbalance due to its value larger than 0.1 [39, 40].

After obtaining the covariate matrix $\varvec{X}=(\varvec{X}_1,\dots ,\varvec{X}_N)^{\top }$, we generate the response $Y_i$ for each subject $(\varvec{X}_i,T_i)$ from the following model with $\tau _{\textrm{SATE}}=\tau _{\textrm{PATE}}=\tau$,

$$\begin{aligned} Y_i = g(\varvec{X}_i) + \tau T_i + \varepsilon _i,\quad \varepsilon _i\sim N(0,1), \end{aligned}$$

where we consider three different forms for the response surface function $g(\varvec{X}_i)$,

$$\begin{aligned} g_{\textrm{L}}(\varvec{X}_i)= & {} 3.5X_{i1}+4.5X_{i3}+1.5X_{i5}+2.5X_{i7}, \quad \quad \quad \quad \quad (\textrm{Linear})\\ g_{\textrm{I}}(\varvec{X}_i)= & {} g_{\textrm{L}}(\varvec{X}_i)+2.5\textrm{sign}(X_{i1})\sqrt{|X_{i1}|} + 2.5X_{i3}X_{i7} ,\quad (\textrm{Interaction})\\ g_{\textrm{P}}(\varvec{X}_i)= & {} g_{\textrm{I}}(\varvec{X}_i)+5.5X_{i3}^2-4.5X_{i1}X_{i3}^3.\quad \ \quad \quad \quad \quad \quad \quad \quad (\textrm{Polynomial}) \end{aligned}$$

Therefore, we can obtain three responses for the *i*th sample under different degrees of nonlinearity. The three types of responses represent the complex response surfaces in real situations, and partial inclusion of covariates in the response functions mimics the fact that not all covariates have an influence on the outcome in practice [38]. The first function $g_{\textrm{L}}(\varvec{X}_i)$ represents a linear surface, whereas the other two functions $g_{\textrm{I}}(\varvec{X}_i)$ and $g_{\textrm{P}}(\varvec{X}_i)$ incrementally consider the nonlinear interactions and higher-order polynomial features. The additive treatment effect is fixed as $\tau =1$ in all situations.

Through the above procedure, we can obtain a dataset including a covariate matrix, a treatment indicator vector, and three response vectors corresponding to three response surface functions for a given ratio *r* and a covariate scenario. We further standardize all covariates to have a unit variance and a zero mean. Following the setting in [41–43], we let the dataset with ratio $r=2$ include the dataset with $r=1$ under the same covariate scenario to correlate different datasets, which can increase the precision of comparisons and save the number of randomly generated datasets. We replicate 200 datasets for all combinations of the ratio *r* and covariate scenario to evaluate different methods.

We first compare the proposed QReR with the original rerandomization (ReR) in terms of the covariate balance and estimation for $\tau _{\textrm{SATE}}$, which reveals how well QReR approximates ReR. For the covariate balance, we consider the similarity between the distributions of the unweighted and weighted covariate mean differences, i.e., $\varvec{\delta }(\widetilde{\varvec{T}})$ and $\varvec{\delta }(\widetilde{\varvec{W}})$. We report the average Kolmogorov–Smirnov (KS) statistics and the corresponding average *p*-values of the mean differences across each covariate, which are based on 1000 weight vectors and acceptable treatment allocations generated by QReR and ReR, respectively.

For the estimation of treatment effect, QReR uses both the observed covariates and responses for inference, whereas we perform ReR on the observed covariates and regenerate an acceptable allocation vector and corresponding responses. Regarding the metrics of assessment, the empirical bias and root mean square error (RMSE) are used to evaluate the precision of the estimator, where the mean difference between treated and control responses is chosen as the estimator for ReR as in [15]. Moreover, we report the Monte Carlo standard errors (MCSEs) for the above performance measurements of simulations (average KS statistics, average *p*-values, bias and RMSE) [44, 45], which evaluate the overall adequacy of simulations with finite repetitions under different random-number seeds.

We consider three different levels of acceptance probability for ReR and QReR, i.e., $p_a=0.1,0.5,1$. For the other hyper-parameters of QReR in Algorithm 1, we set the iteration numbers as $(N_{\textrm{init}},N_{\textrm{train}},N_{\textrm{stop}})=(500,5000,15)$ for network initialization, training and early-stopping, and the batch sizes for training are specified as $(B,B_{\textrm{init}},B_{\textrm{stop}},B_{\textrm{loss}})=(512,1000,1000,10000)$. We use the Adam optimizer [46] with default parameters to conduct the stochastic gradient descent algorithm. The regularization coefficients $\lambda _1$ and $\lambda _2$ are both fixed as 1. During the inference, the QReR estimator $\hat{\tau }_M$ is calculated based on $M=1000$ weight vectors, denoted by $\textrm{QReR}_{\textrm{M}}$. In addition, we compute the estimator using a single weight vector ($M=1$) denoted by $\textrm{QReR}_{\textrm{S}}$ to compare with ReR and show the advantage of ensembling multiple weight vectors.

We further study the performance of QReR on inferring $\tau _{\textrm{PATE}}$ in comparison with several popular balancing approaches. We consider the propensity score matching (PSM) provided by the R package Matching [47], optimal full matching (FM) in the MatchIt package [48, 49], inverse probability weighting (IPW) using the propensity score (Eq. 7 in [50]), entropy balancing (EBAL) proposed by [12], stable balancing weights (SBW) in [13] and empirical balancing calibration weighting (EBCW) based on the package ATE [14], where EBAL, SBW and EBCW are non-parametric reweighting algorithms. We use the R package WeightIt [37] to conduct SBW as well as EBAL, and the tolerance parameter of SBW is specified as 0.01. The propensity scores are estimated using the logistic regression in the relevant benchmarks, including PSM, FM and IPW. No further covariate adjustment is applied when estimating $\tau _{\textrm{PATE}}$ after matching or reweighting. For QReR, we keep the same settings of hyper-parameters in the $\tau _{\textrm{SATE}}$ estimation except that we only approximate the most stringent ReR with $p_a=0.1$. Similar to [11–14], we focus on the bias and RMSE in the estimation of $\tau _{\textrm{PATE}}$ when evaluating different methods.

### Simulation results

**Fig. 1:**
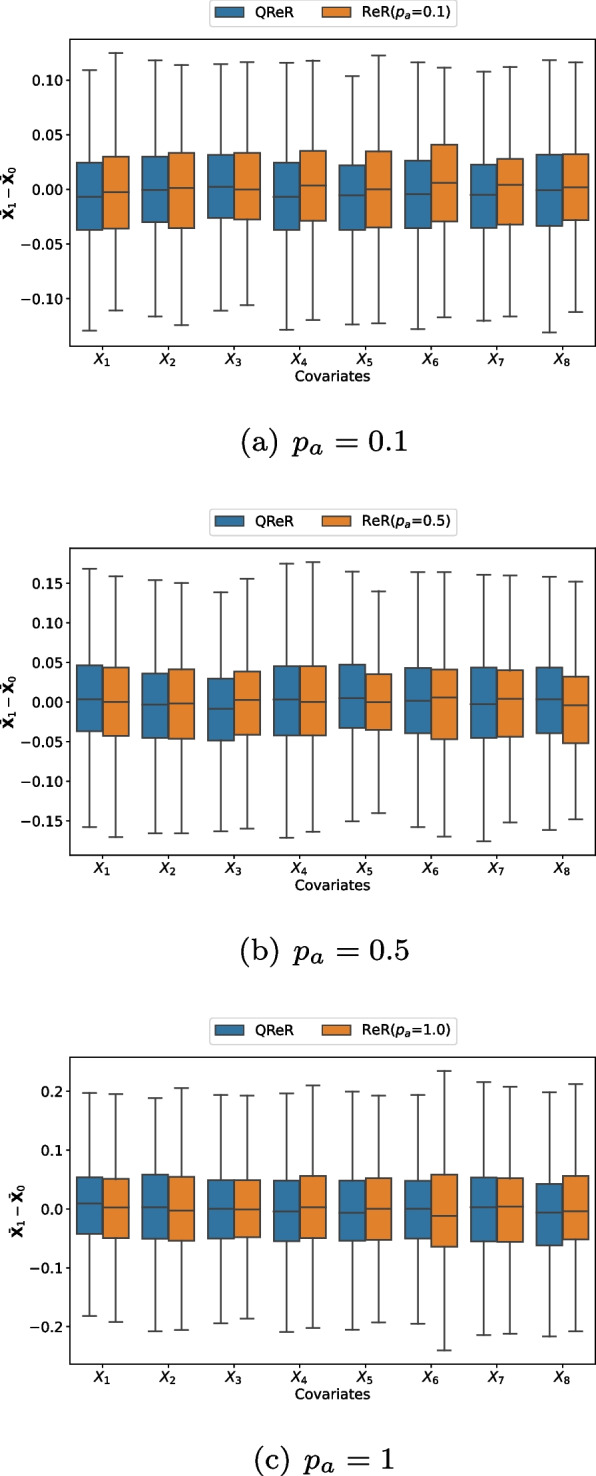
An illustrative example for the (weighted) mean differences of all covariates between quasi-rerandomization (QReR) and rerandomization (ReR) with $p_a=0.1, 0.5, 1$. The boxplots are based on 1000 acceptable ReR allocations $\widetilde{\varvec{T}}$ and transformed weights $\widetilde{\varvec{W}}$ under a simulated dataset from Scenario 1 with $r=N_0/N_1=2$

Table 1 shows that QReR can approximate ReR well in terms of covariate balance. First, the average MCSEs have small values for both the average KS statistics and *p*-values, which implies that replications of 200 are adequate. For different combinations of *r* and scenarios, the average KS statistics are all small with the corresponding average *p*-values larger than 0.1, indicating that the weighted mean differences of QReR for each covariate share similar distributions to the counterparts in ReR. Furthermore, the KS statistics are larger for smaller $p_a$ (more stringent), which implies that it is more difficult for QReR to reconstruct ReR when the criterion of covariate balance is more stringent. It may result from the fact that the distribution of $\varvec{\delta }(\varvec{T})$ is more concentrated around zero for small $p_a$ and thus is more difficult to approximate. For a more intuitive illustration, we draw the boxplots to visualize the covariate mean differences of a representative case for ReR and QReR with $p_a=0.1$ in Fig. 1, where covariates are generated under Scenario 1 with $r=2$. We observe that the paired boxplots of QReR and ReR generally exhibit similar shapes especially for the continuous covariates and a large value of $p_a$. The medians in the paired boxplots are close, indicating that QReR and ReR have similar covariate balance, whereas other quantile points further show that the covariate mean difference of QReR displays a similar variation to that of ReR.

**Table 1:** The average Kolmogorov–Smirnov (KS) statistics and average *p*-values of covariate mean differences across all covariates between 1000 weighted and balanced datasets generated respectively by quasi-rerandomization and rerandomization. The average Monte Carlo standard errors (MCSEs) are 0.001 and 0.005 for the average KS statistics and the average *p*-values, respectively

Scenario	$p_a$	$r=1$		$r=2$	
		KS	*p*-value	KS	*p*-value
1	0.1	0.083	0.124	0.081	0.129
	0.5	0.073	0.150	0.071	0.148
	1	0.068	0.161	0.067	0.157
2	0.1	0.084	0.133	0.080	0.133
	0.5	0.075	0.135	0.071	0.160
	1	0.069	0.151	0.066	0.160
3	0.1	0.084	0.127	0.082	0.124
	0.5	0.074	0.144	0.072	0.142
	1	0.068	0.154	0.067	0.157

Tables 2 and 3 show the estimation performance of QReR in comparison with ReR. We first observe that the average MCSEs have relatively larger values for the NonLinear (Polynomial) model, because it is more difficult to balance covariates and thus leads to larger variations under the complex surface function. For QReR, we simultaneously consider two estimators $\textrm{QReR}_{\textrm{M}}$ and $\textrm{QReR}_{\textrm{S}}$ based on (13). When the outcome is generated from Linear or Nonlinear (Interaction) models, QReR yields comparable bias and RMSE to ReR. However, we observe much larger bias and RMSE under QReR for the response surface of Nonlinear (Polynomial) and covariates from Scenarios 2 and 3. The results under Nonlinear (Interaction) indicate that QReR for the observational data can demonstrate similar performance to rerandomized experiments even in the presence of unobserved nonlinear covariates. This could be explained by the MMD loss being capable of incorporating some nonlinear information of covariates by taking various orders of moments for $\varvec{\Delta }(\widetilde{\varvec{T}})$ into account. In contrast, ReR inherently improves the covariate balance of any unobserved covariates due to the nature of randomization. Additionally, we find that QReR mimics ReR by delivering smaller RMSEs using a smaller acceptance probability $p_a$ under various situations, which results from smaller values of $\varvec{\delta }(\widetilde{\varvec{T}})$’s entries in (6) due to more balanced covariates in ReR. The RMSEs of QReR and ReR are also smaller for $r=2$ in contrast to $r=1$, because more observations are provided. Concerning $\textrm{QReR}_{\textrm{M}}$ and $\textrm{QReR}_{\textrm{S}}$, we find that the simpler estimator $\textrm{QReR}_{\textrm{S}}$ demonstrates more similar values of bias and RMSE to ReR, which stems from the similar covariate balance between QReR and ReR. The similarity also reflects that the single weight vector from QReR can approximate the inference properties of the acceptable allocation in ReR. The estimator $\textrm{QReR}_{\textrm{M}}$ generally has smaller RMSE because it ensembles multiple $\textrm{QReR}_{\textrm{S}}$’s via taking the average, which shows the advantage of generating diverse weight vectors.

**Table 2:** The bias and RMSE for $\tau _{\textrm{SATE}}$ under quasi-rerandomization (QReR) and rerandomization (ReR) under different combinations of the acceptance probability $p_a$, covariate scenarios and response surfaces when $r=N_0/N_1=1$. The average Monte Carlo standard errors (MCSEs) of bias are 0.03, 0.04 and 0.31 and those of RMSE are 0.02, 0.03 and 0.31 for Linear, NonLinear (Interaction) and NonLinear (Polynomial) models, respectively

Response	$p_a$	Method	Scenario 1		Scenario 2		Scenario 3	
			Bias	RMSE	Bias	RMSE	Bias	RMSE
Linear	0.1	$\textrm{QReR}_{\textrm{S}}$	-0.002	0.35	0.005	0.44	0.020	0.42
		$\textrm{QReR}_{\textrm{M}}$	-0.022	0.12	-0.032	0.12	-0.028	0.12
		ReR	0.004	0.31	0.001	0.39	0.003	0.41
	0.5	$\textrm{QReR}_{\textrm{S}}$	-0.053	0.47	-0.059	0.60	-0.067	0.66
		$\textrm{QReR}_{\textrm{M}}$	-0.037	0.12	-0.059	0.13	-0.058	0.13
		ReR	0.029	0.45	0.038	0.53	-0.001	0.56
	1	$\textrm{QReR}_{\textrm{S}}$	-0.035	0.60	-0.017	0.67	-0.062	0.76
		$\textrm{QReR}_{\textrm{M}}$	-0.054	0.12	-0.064	0.14	-0.060	0.14
		ReR	-0.023	0.57	-0.038	0.68	0.072	0.72
NonLinear	0.1	$\textrm{QReR}_{\textrm{S}}$	-0.017	0.49	-0.060	0.64	-0.071	0.62
(Interaction)		$\textrm{QReR}_{\textrm{M}}$	-0.033	0.19	-0.123	0.24	-0.132	0.25
		ReR	0.014	0.46	0.011	0.54	0.032	0.62
	0.5	$\textrm{QReR}_{\textrm{S}}$	-0.079	0.65	-0.162	0.83	-0.199	0.93
		$\textrm{QReR}_{\textrm{M}}$	-0.055	0.19	-0.148	0.25	-0.165	0.27
		ReR	0.041	0.63	0.053	0.74	-0.020	0.76
	1	$\textrm{QReR}_{\textrm{S}}$	-0.061	0.84	-0.095	0.94	-0.166	1.10
		$\textrm{QReR}_{\textrm{M}}$	-0.075	0.20	-0.148	0.25	-0.152	0.27
		ReR	-0.037	0.80	-0.058	0.95	0.090	1.02
NonLinear	0.1	$\textrm{QReR}_{\textrm{S}}$	0.078	2.31	5.001	7.46	-5.931	7.69
(Polynomial)		$\textrm{QReR}_{\textrm{M}}$	0.025	2.04	4.869	7.02	-6.118	7.58
		ReR	0.030	2.03	-0.571	5.75	0.368	5.77
	0.5	$\textrm{QReR}_{\textrm{S}}$	-0.051	2.54	4.958	7.75	-6.869	8.77
		$\textrm{QReR}_{\textrm{M}}$	-0.063	2.12	4.861	7.23	-6.551	7.98
		ReR	-0.007	1.94	-0.289	5.60	0.119	6.16
	1	$\textrm{QReR}_{\textrm{S}}$	-0.051	2.65	4.774	7.54	-7.008	8.77
		$\textrm{QReR}_{\textrm{M}}$	-0.071	2.15	4.703	7.05	-7.229	8.61
		ReR	-0.048	2.07	-0.029	6.03	-0.020	6.48

$\textrm{QReR}_{\textrm{S}}$: using a single random weight vector generated by our model to conduct inference; $\textrm{QReR}_{\textrm{M}}$: using the average weight vector based on $M=1000$ random weights

**Table 3:** The bias and RMSE for $\tau _{\textrm{SATE}}$ under quasi-rerandomization (QReR) and rerandomization (ReR) under different combinations of the acceptance probability $p_a$, covariate scenarios and response surfaces when $r=N_0/N_1=2$. The average Monte Carlo standard errors (MCSEs) of bias are 0.02, 0.03 and 0.26 and those of RMSE are 0.02, 0.02 and 0.30 for Linear, NonLinear (Interaction) and NonLinear (Polynomial) models, respectively

Response	$p_a$	Method	Scenario 1		Scenario 2		Scenario 3	
			Bias	RMSE	Bias	RMSE	Bias	RMSE
Linear	0.1	$\textrm{QReR}_{\textrm{S}}$	-0.032	0.31	-0.051	0.36	0.011	0.35
		$\textrm{QReR}_{\textrm{M}}$	-0.017	0.10	-0.018	0.10	-0.013	0.10
		ReR	-0.001	0.27	0.019	0.29	0.031	0.32
	0.5	$\textrm{QReR}_{\textrm{S}}$	-0.018	0.40	-0.016	0.49	-0.069	0.51
		$\textrm{QReR}_{\textrm{M}}$	-0.034	0.11	-0.029	0.10	-0.036	0.11
		ReR	0.026	0.37	0.044	0.42	-0.020	0.44
	1	$\textrm{QReR}_{\textrm{S}}$	-0.031	0.49	-0.053	0.55	0.012	0.53
		$\textrm{QReR}_{\textrm{M}}$	-0.037	0.10	-0.044	0.11	-0.047	0.11
		ReR	-0.022	0.48	-0.015	0.55	-0.067	0.57
NonLinear	0.1	$\textrm{QReR}_{\textrm{S}}$	-0.102	0.44	-0.207	0.56	-0.121	0.51
(Interaction)		$\textrm{QReR}_{\textrm{M}}$	-0.081	0.18	-0.143	0.23	-0.146	0.25
		ReR	0.004	0.40	0.030	0.43	0.042	0.46
	0.5	$\textrm{QReR}_{\textrm{S}}$	-0.089	0.57	-0.122	0.69	-0.201	0.76
		$\textrm{QReR}_{\textrm{M}}$	-0.097	0.19	-0.149	0.24	-0.167	0.26
		ReR	0.038	0.52	0.056	0.59	-0.030	0.61
	1	$\textrm{QReR}_{\textrm{S}}$	-0.086	0.70	-0.167	0.80	-0.086	0.74
		$\textrm{QReR}_{\textrm{M}}$	-0.092	0.19	-0.157	0.25	-0.174	0.27
		ReR	-0.018	0.65	-0.009	0.74	-0.085	0.79
NonLinear	0.1	$\textrm{QReR}_{\textrm{S}}$	-0.281	1.76	4.671	7.17	-4.674	6.52
(Polynomial)		$\textrm{QReR}_{\textrm{M}}$	-0.267	1.63	4.819	6.82	-4.817	6.25
		ReR	0.100	1.70	0.094	4.28	-0.323	4.60
	0.5	$\textrm{QReR}_{\textrm{S}}$	-0.298	1.93	5.055	7.28	-5.632	7.32
		$\textrm{QReR}_{\textrm{M}}$	-0.308	1.71	4.840	6.91	-5.519	6.88
		ReR	0.114	1.67	0.238	4.24	-0.382	5.06
	1	$\textrm{QReR}_{\textrm{S}}$	-0.294	1.97	4.872	7.31	-5.437	7.32
		$\textrm{QReR}_{\textrm{M}}$	-0.310	1.74	4.859	7.04	-5.925	7.32
		ReR	0.030	1.74	0.325	4.31	0.012	4.91

The point estimator for $\tau _{\textrm{SATE}}$ has the same form of $\tau _{\textrm{PATE}}$ so that we only compare $\textrm{QReR}_{\textrm{M}}$ with other balancing algorithms in terms of bias and RMSE as shown by Table 4. In most cases, our approach has RMSE and bias as small as other non-parametric weighting approaches including SBW, EBAL and EBCW, and outperforms the parametric weighting method IPW as well as other matching methods such as PSM and FM. In addition, we find that SBW, EBAL and EBCW perform better than $\textrm{QReR}_{\textrm{M}}$ and the propensity score methods in terms of bias under Linear and Nonlinear (Interaction) models. Furthermore, $\textrm{QReR}_{\textrm{M}}$ demonstrates an evident advantage when the response is generated from a highly nonlinear response surface with heterogeneous covariate distributions. It shows that despite being inferior to ReR, our method performs better in the presence of nonlinear covariates relative to other balancing approaches. Moreover, unlike those benchmarks that rely on the balanced covariates without clear hypothetical randomized experiments, our estimator is pillared by the weight vectors that directly approximate the rerandomized experiment. Therefore, our method can incorporate rerandomization to balance covariates and simultaneously yield precise estimation of the treatment effect.

**Table 4:** The bias and RMSE for $\tau _{\textrm{PATE}}$ under quasi-rerandomization (QReR) and other balancing methods under different combinations of the covariate scenarios, response surfaces and ratios ($r=N_0/N_1$). The average Monte Carlo standard errors (MCSEs) of bias are 0.02, 0.03 and 0.40 and those of RMSE are 0.02, 0.02 and 0.77 for Linear, NonLinear (Interaction) and NonLinear (Polynomial) models, respectively

*r*	Response	Method	Scenario 1		Scenario 2		Scenario 3	
			$\textrm{Bias}$	RMSE	$\textrm{Bias}$	RMSE	$\textrm{Bias}$	RMSE
1	Linear	IPW	0.040	0.42	0.217	0.55	0.179	0.50
		PSM	0.162	0.42	0.247	0.57	0.365	0.67
		FM	0.156	0.40	0.280	0.57	0.378	0.66
		EBAL	-0.002	0.11	-0.003	0.11	-0.005	0.11
		SBW	-0.003	0.10	-0.004	0.11	-0.005	0.11
		EBCW	-0.002	0.11	-0.003	0.11	-0.004	0.11
		$\textrm{QReR}_{\textrm{M}}$	-0.022	0.12	-0.031	0.12	-0.029	0.12
	Nonlinear	IPW	0.077	0.58	0.290	0.72	0.245	0.64
	(Interaction)	PSM	0.245	0.61	0.241	0.81	0.423	0.95
		FM	0.235	0.59	0.292	0.80	0.440	0.93
		EBAL	0.012	0.19	-0.029	0.22	-0.041	0.21
		SBW	0.011	0.19	-0.042	0.22	-0.056	0.22
		EBCW	0.012	0.19	-0.029	0.22	-0.040	0.21
		$\textrm{QReR}_{\textrm{M}}$	-0.033	0.19	-0.123	0.24	-0.133	0.26
	Nonlinear	IPW	0.247	3.56	5.333	11.91	-10.193	14.00
	(Polynomial)	PSM	0.097	3.54	5.217	9.40	-8.803	10.90
		FM	0.124	3.53	5.134	9.29	-8.872	11.00
		EBAL	0.092	2.72	4.925	8.51	-8.850	10.62
		SBW	0.119	2.55	5.419	8.54	-7.836	9.52
		EBCW	0.092	2.72	4.925	8.51	-8.850	10.62
		$\textrm{QReR}_{\textrm{M}}$	0.025	2.04	4.875	7.01	-6.112	7.56
2	Linear	IPW	0.024	0.35	-0.565	0.96	-1.154	1.42
		PSM	0.085	0.41	-0.032	0.49	-0.311	0.58
		FM	0.088	0.40	-0.035	0.48	-0.302	0.56
		EBAL	0.002	0.09	0.002	0.09	0.001	0.09
		SBW	0.001	0.09	0.001	0.09	0.000	0.09
		EBCW	0.002	0.09	0.002	0.09	0.001	0.09
		$\textrm{QReR}_{\textrm{M}}$	-0.017	0.10	-0.017	0.10	-0.013	0.10
	Nonlinear	IPW	0.049	0.46	-0.766	1.23	-1.611	1.91
	(Interaction)	PSM	0.130	0.56	-0.174	0.68	-0.603	0.91
		FM	0.132	0.54	-0.179	0.67	-0.595	0.88
		EBAL	0.006	0.17	-0.023	0.20	-0.032	0.20
		SBW	-0.052	0.17	-0.104	0.22	-0.121	0.23
		EBCW	0.006	0.17	-0.023	0.20	-0.032	0.20
		$\textrm{QReR}_{\textrm{M}}$	-0.080	0.18	-0.142	0.23	-0.145	0.25
	Nonlinear	IPW	0.060	3.22	4.734	14.91	-12.453	18.48
	(Polynomial)	PSM	-0.105	2.72	5.280	9.50	-8.156	10.14
		FM	-0.064	2.66	5.230	9.45	-8.279	10.20
		EBAL	-0.056	2.20	5.098	8.74	-8.151	10.08
		SBW	-0.069	2.09	5.678	8.83	-6.955	8.77
		EBCW	-0.056	2.20	5.098	8.74	-8.152	10.08
		$\textrm{QReR}_{\textrm{M}}$	-0.261	1.63	4.810	6.81	-4.825	6.26

IPW: inverse probability weighting using propensity scores; PSM: propensity score matching; FM: optimal full matching; EBAL: entropy balancing; SBW: stable balancing weights; EBCW: empirical balancing calibration weighting; and $\textrm{QReR}_{\textrm{M}}$: quasi-rerandomization using average weight vector with acceptance probability $p_a=0.1$

### Real application

We demonstrate the application of our proposed QReR on semi-synthetic data [51, 52], which consist of real and imbalanced covariates with allocations and simulated responses. The covariates were collected from the Infant Health and Development Program (IHDP), which targeted the low-birth-weight and premature infants. There were 19 binary covariates and 6 continuous covariates for each participant. High-quality childcare and professional home visits were provided for the treatment group, and the infants’ cognitive test score was the outcome of interest. There were 747 observations with 139 and 608 units for the treatment and control groups, respectively. Given the real covariates and allocations, Hill [51] simulated responses from various response surface functions to obtain the true treatment effect and thus evaluate different methods.

Shalit et al. [52] publicly provided 100 such datasets^1^ with different average treatment effects, each of which includes the potential outcomes $\{(Y_{i}(0), Y_{i}(1))\}_{i=1}^{747}$ with their expectations $\left\{ (\mathbb {E}\{Y_{i}(1)|X_i\}, \mathbb {E}\{Y_{i}(0)|X_i\})\right\} _{i=1}^{747}$, the treatment indicator vector $\varvec{T}\in \mathbb {R}^{747}$ with $\sum _{i=1}^NT_i=139$ and the same covariate matrix $\varvec{X}\in \mathbb {R}^{747\times 25}$ using all 25 covariates. Moreover, these 100 datasets have heterogeneous individual treatment effects, i.e., both $Y_{i}(1)-Y_{i}(0)$ and its expectation $\mathbb {E}\{Y_{i}(1)|X_i\} - \mathbb {E}\{Y_{i}(0)|X_i\}$ have different values for $i=1,\dots , N$. We further standardize all covariates to have zero means and unit variances. Based on the potential outcomes, we can easily obtain the finite sample average treatment effect $\tau _{\textrm{SATE}}$ for each dataset, so that QReR can be similarly compared with ReR in terms of the bias and RMSE. We set the true population average treatment effect $\tau _{\textrm{PATE}}=\sum _{i=1}^N\left[ \mathbb {E}\{Y_{i}(1)|X_i\}-\mathbb {E}\{Y_{i}(0)|X_i\}\right] /N$ following [52], and calculate the corresponding bias and RMSE for QReR and other balancing techniques. We use the same parameter settings in the simulations for various balancing approaches in analysis of IHDP datasets.

**Fig. 2:**
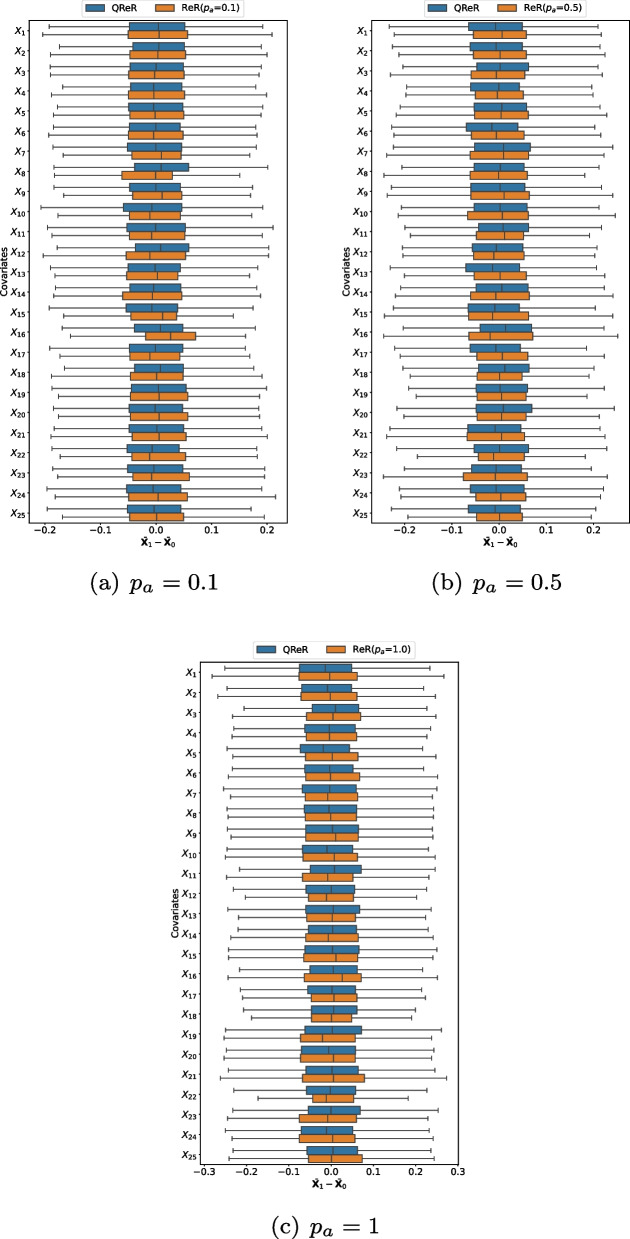
The (weighted) mean differences of all covariates between quasi-rerandomization (QReR) and rerandomization (ReR) with $p_a=0.1, 0.5, 1$ on the covariates of IHDP datasets. The boxplots are based on 1000 acceptable ReR allocations $\widetilde{\varvec{T}}$ and transformed weights $\widetilde{\varvec{W}}$. The first six covariates are continuous, whereas the last nineteen covariates are binary

In Fig. 2, we present the covariate mean differences between QReR and ReR for continuous ($X_1,\dots , X_6$) and binary ($X_7,\dots , X_{25}$) covariates under different values of $p_a$. It shows that $\varvec{\delta }(\widetilde{\varvec{W}})$ of QReR generally has similar distributions to $\varvec{\delta }(\widetilde{\varvec{T}})$ of ReR on the 25 covariates, particularly for the continuous ones, and thus QReR well reconstructs ReR in terms of the covariate mean differences. Furthermore, we observe that QReR can better approximate ReR when $p_a$ increases, which is consistent with the findings from the simulations.

From the results in Table 5, we find that QReR also yields comparable bias and RMSE to ReR, and $\textrm{QReR}_{\textrm{M}}$ performs better than $\textrm{QReR}_{\textrm{S}}$ under the heterogeneous treatment effects, which strengthens the tendency of using multiple weight vectors. Table 6 shows that $\textrm{QReR}_{\textrm{M}}$ achieves the smallest bias in contrast with other benchmarks. Its RMSE is much smaller than the matching approaches, but slightly worse than SBW, EBAL and EBCW due to a trade-off on the bias. This hence shows that our proposed approach is still advantageous in estimating $\tau _{\textrm{PATE}}$ under the context of real covariates and heterogeneous individual treatment effects, and thus can be adopted broadly in the real applications.

**Table 5:** The bias and RMSE for the sample average treatment effect using quasi-rerandomization (QReR) and rerandomization (ReR) on the IHDP datasets, under the acceptance probability $p_a= 0.1, 0.5, 1$

$p_a$	Method	Bias	RMSE
0.1	$\textrm{QReR}_{\textrm{S}}$	-0.068	0.29
	$\textrm{QReR}_{\textrm{M}}$	-0.016	0.19
	ReR	-0.008	0.21
0.5	$\textrm{QReR}_{\textrm{S}}$	0.019	0.34
	$\textrm{QReR}_{\textrm{M}}$	-0.035	0.19
	ReR	0.017	0.18
1	$\textrm{QReR}_{\textrm{S}}$	-0.104	0.34
	$\textrm{QReR}_{\textrm{M}}$	-0.031	0.18
	ReR	-0.022	0.25

**Table 6:** The bias and RMSE for the population average treatment effect using various balancing methods on the IHDP datasets. The quasi-rerandomization (QReR) reconstructs the rerandomization under the acceptance probability $p_a=0.1$

Method	Bias	RMSE
IPW	-0.853	1.143
PSM	0.065	0.231
FM	-0.101	0.295
EBAL	-0.028	0.184
SBW	-0.030	0.180
EBCW	-0.028	0.184
$\textrm{QReR}_{\textrm{M}} (p_a=0.1)$	-0.021	0.191

## Conclusions

We propose a novel balancing technique, named quasi-rerandomization, for observational studies, which incorporates the covariate balance from rerandomization into the observational data via reweighting. The weights obtained from our method can be conveniently combined with weighted point estimators to perform the subsequent inference for both finite-sample and population treatment effects. We empirically show that our method can well approximate the rerandomized experiments in terms of improving the covariate balance and the precision of treatment effect estimation. Furthermore, our approach demonstrates competitive performance compared with other weighting and matching methods. Possible extensions may modify our algorithm to approximate other types of randomized experiments, such as block randomization. One may also explore whether the Bayesian framework can be leveraged to generate the weights that can reconstruct rerandomization in terms of covariate balance. The codes and datasets for the simulations and real application can be found at https://github.com/BobZhangHT/QReR.

## Data Availability

The data used in the manuscript are either simulated or publicly available, which can be found at https://github.com/BobZhangHT/QReR.

## Footnotes

1 The datasets can be downloaded from https://www.fredjo.com by merging two subsets IHDP-100 (train) and IHDP-100 (test).
